# Molecular Mechanisms Involved in the B Cell Growth and Clonogenic Activity of HIV-1 Matrix Protein p17 Variants

**DOI:** 10.3390/v16071048

**Published:** 2024-06-28

**Authors:** Pasqualina D’Ursi, Alessandro Rondina, Alberto Zani, Matteo Uggeri, Serena Messali, Arnaldo Caruso, Francesca Caccuri

**Affiliations:** 1 Institute of Technologies in Biomedicine, National Research Council, 20090 Segrate, Italy; 2 Section of Microbiology, Department of Molecular and Translational Medicine, University of Brescia, 25123 Brescia, Italymatteo.uggeri@light-center.it (M.U.);; 3 Lifescience Innovation Good Healthcare Technology—LIGHT s.c.ar.l., 25123 Brescia, Italy; 4 Centre for Advanced Medical and Pharmaceutical Research, “George Emil Palade” University of Medicine, Pharmacy, Science and Technology, 540142 Targu Mures, Romania

**Keywords:** HIV-1 matrix protein p17 variants, AIDS-related lymphoma, accelerate molecular dynamic, gain of function, hydrophobic core

## Abstract

The human immunodeficiency virus (HIV-1) matrix protein p17 (p17) is released from infected cells as a protein capable of deregulating the biological activity of different cells. P17 variants (vp17s), more frequently detected in the plasma of HIV-1^+^ patients with rather than without lymphoma and characterized by amino acids insertions in their C-terminal region, were found to trigger B cell growth and clonogenicity. Vp17s endowed with B-cell-growth-promoting activity are drastically destabilized, whereas, in a properly folded state, reference p17 (refp17) does not exert any biological activity on B cell growth and clonogenicity. However, misfolding of refp17 is necessary to expose a masked functional epitope, interacting with the protease-activated receptor 1 (PAR-1), endowed with B cell clonogenicity. Indeed, it is worth noting that changes in the secondary structure can strongly impact the function of a protein. Here, we performed computational studies to show that the gain of function of vp17s is linked to dramatic conformational changes due to structural modification in the secondary-structure elements and in the rearrangement of the hydrogen bond (H-bond) network. In particular, all clonogenic vp17s showed the disengagement of two critical residues, namely Trp16 and Tyr29, from their hydrophobic core. Biological data showed that the mutation of Trp16 and Tyr29 to Ala in the refp17 backbone, alone or in combination, resulted in a protein endowed with B cell clonogenic activity. These data show the pivotal role of the hydrophobic component in maintaining refp17 stability and identify a novel potential therapeutic target to counteract vp17-driven lymphomagenesis in HIV-1^+^ patients.

## 1. Introduction

The human immunodeficiency virus (HIV-1) matrix protein p17 (p17) is a Gag-encoded 132-amino acids (aa)-long protein known for its pivotal role in the virus life cycle [1]. The protein has been found to be expressed at the extracellular level in different tissues and organs [2], and it accumulates in the lymph nodes of patients even during pharmacological control of viral replication [3]. Recently, the molecular mechanism responsible for protein secretion from both HIV-1-infected or latently infected cells has been unraveled [4], thus explaining the presence of virion-free p17 in the tissue microenvironment. Extracellularly, p17, derived from the clade B (BH10; refp17) virus, was found to deregulate the biological activity of relevant cell populations in the context of HIV-1 pathogenesis [5].

The viral protein is characterized by a globular domain formed by five α-helices (Hs), three of which (H1, H2, and H3) fold around the central and buried H4, while H5 is projected from the globular domain [6]. Differently from refp17, p17 variants (vp17s), characterized by aa insertions at different positions in the H5, were found to be more expressed in HIV-1-seropositive (HIV^+^) patients with lymphomas as compared to patients without lymphoma [7]. Interestingly, vp17s were found to be capable of triggering B cell growth and clonogenicity [8], suggesting a role for these proteins in HIV-1-related lymphomagenesis [9,10]. This gain of function is linked to dramatic conformational changes in the viral protein due to structural modification in the secondary-structure elements and in the rearrangement of the hydrogen bond (H-bond) network. 

Misfolding of refp17 was found to be necessary to expose a functional epitope [8], whose B-cell-growth-promoting activity is exerted upon interaction with the protease-activated receptor 1 (PAR-1) [11]. As expected, B cell clonogenic vp17s derived from HIV^+^ patients with lymphoma were also found to be misfolded compared with refp17. Further exploration of structure–function relationships by dissecting refp17 has led to the identification of the functional epitope in the N-terminal region of the viral protein. A synthetic peptide, namely F1, spanning from aa 2 to 21, was found to exert B cell clonogenic activity [12]. The F1 peptide is composed of a hydrophobic (aa 2–11) and a hydrophilic region (aa 12–21). An alanine scanning approach for peptide F1 was performed to investigate the effect of each residue on its biological activity. The hydrophobic region of peptide F1 was excluded from the clonogenic activity of the peptide, while the charged residues Arg 15, Lys 18, and Arg 20 were found responsible for the activity of peptide F1 on B cells [13].

The impact of the hydrophobic aspect of a protein on the three-dimensional arrangement of residues is extensively acknowledged in the literature [14,15,16]. Single mutations, particularly those altering the level of hydrophobicity, result in different residues participating in the formation of the hydrophobic core, which arises from a synergistic effect in which the entire chain is involved. The status of individual residues, in fact, is determined by their intrinsic propensity to contribute to the core formation, while also being influenced by the surrounding environment [17,18]. 

Given the current limited understanding of vp17s behavior, our approach has employed computational methods to unravel the mechanism involved in the exposure of the functional epitope. In particular, we investigated the pivotal role of the hydrophobic component, which seems to exert a fundamental influence. By focusing on specific residues, here, we elucidate their significance in maintaining protein stability and highlight how exposure of the functional epitope for B cell growth and clonogenicity occurs in the misfolded protein.

## 2. Materials and Methods

### 2.1. Modeling of refp17 and vp17s

1TAM and 2HMX p17 structures were retrieved from the PDB database [19] and used as templates for the modeling of refp17; vp17s derived from HIV^+^ patients, namely NHL-a101, NHL-a104, and vp17c2 [7]; or refp17 displaying W16A and Y29A mutations (p17^W16A+Y29A^). The proteins were modeled using the MODELLER program (version 10.5) [20]. A comparative multi-template modeling approach was applied to build a complete structure of the proteins using both previously mentioned template structures. Structural assessment of the predicted protein models was performed via Ramachandran plot using the PROCHECK software (version 3.5.4) [21] and qualitative model energy analysis (QMEANDisCo) [22]. The model demonstrating the highest structural qualities was then selected to build a simulation system.

### 2.2. Classic Molecular Dynamics

Classic molecular dynamic (cMD) simulations were performed with the Amber18 Molecular Dynamics package using the ff14SB force fields [23,24]. The 3D structures were solvated with TIP3P water models in octahedral boxes extending 10 Å from the protein surface. The system was then neutralized with counterions at a concentration of 150 mM [25]. Energy minimization of the system was carried out in four consecutive steps, each of which consists of 5000 steps, using the steepest descent method and decreasing restraint in order to allow the system to stabilize gradually. In the first minimization step, all the atoms, except for hydrogens, were restrained at 2.0 Kcal/mol; in the second minimization, restraints were lowered to 1.0 Kcal/mol and removed for water molecules; then, in the third minimization step, the restraints were further decreased to 0.5 Kcal/mol; while in the last minimization step, all restraints were removed. The system was then heated from 0 K to 300 K at a constant volume using a Langevin thermostat, with the protein atoms restrained at 1.0 Kcal/mol for 200,000 steps. Afterward, the system was equilibrated at constant pressure for 3 ns. Finally, each system was subjected to a cMD simulation of 100 ns without restraints, with the cutoff for non-bonded interactions set at 8 Å.

### 2.3. Accelerated Molecular Dynamics

Accelerated molecular dynamic (aMD) simulation is a technique aimed at increasing the normal conformational sampling of macro and micro molecules through the addition of a non-negative boost potential, capable of applying an energy boost to the system. Average potential energy (Ep) and dihedral energies (Ed) are necessary as reference energies to calculate the appropriate boost factor to apply [26,27]. 

To better explore the conformational dynamics of proteins, a “dual-boost” aMD was performed. Average Ep and Ed values for each system were obtained from 100 ns of cMD simulations and used to calculate the EthreshD (Ed), alphaD (αD), EthreshP (Ep), and alphaP (αP) values needed for aMD (Table S1), as described by [28]. All systems were finally simulated for 700 ns. The simulation of the NHL-a101 vp17 was extended to 1 μs to further explore the energy landscape, allowing the system to reach a state where observable changes become possible.

### 2.4. Molecular Dynamics Data Analysis

CPPTRAJ of AMBER18 was used to analyze the MD simulation results. The hydrogen bond network was characterized by calculating the H-bond interactions, considering only those present for more than 75% during the simulation [29], while the hydrophobic interactions were characterized using the protein interactions calculator (PIC) [30]. For this purpose, 3D structures of proteins were extracted from aMD every 1000 frames; each structure was then used as input for the PIC software. Output data were finally combined, and statistics were calculated. We assessed hydrophobic interactions in the refp17 protein that persisted for more than 60% of the simulation. These interactions were then compared to those assessed in the vp17s. 

To investigate whether Trp16 and Tyr29, two residues of the hydrophobic core of p17, contribute to the impaired activity of the variants, the values of the dihedral angles Φ (phi) and ψ (psi), defined on the backbone atoms, and χn (chi-n), defined on the side chain atoms, were calculated. Atoms of angles phi (C-N-CA-C), psi (N-CA-C-N), and chi1(C-CA-CB-CG) of the Trp16 and Tyr29 and chi2 (CA-CB-CG-CD) of Trp16 were selected for this analysis (Figure S1). 

Principal component analysis (PCA) was performed to reduce the number of dimensions needed systematically to reveal the most important motions in proteins via a decomposition process that filters the observed motions from the largest to the smallest spatial scales. First, the covariance matrix was calculated from simulation data. Rotational and translational motions of the proteins during the MD trajectory were removed by performing the best coordinates fit on a reference and by calculating their root mean square deviation (RMSD). PCs were then obtained by diagonalizing the covariance matrix, thus allowing for the determination of the eigenvalues and eigenvectors of each PC. The analysis was performed on the backbone of the protein to provide a more detailed characterization of essential spatial motions.

### 2.5. Cell Cultures

Human lymphoma B cell line Raji was obtained from American Type Culture Collection (ATCC, Manassas, VA, USA) and cultured in complete medium containing RPMI-1640 medium supplemented with 10% (*v*/*v*) fetal bovine serum (FBS), 1 mM L-glutamine, and 1 mM sodium pyruvate. Cells were maintained at 37 °C in a humidified atmosphere of 5% CO_2_. 

### 2.6. Recombinant Proteins and Monoclonal Antibodies to p17

Purified endotoxin (LPS)-free recombinant viral proteins were produced as previously described [31]. Briefly, the coding sequence of refp17 (aa 1–132) derived from the HIV-1 BH10 isolate was amplified via PCR with specific primers, which allowed us to clone the refp17 sequence into the BamHI site of the prokaryotic expression vector pGEX-2T (GE Healthcare, Chicago, IL, USA). The vp17 NHL-a101, obtained from the lymphoma biopsy of an HIV-1-infected patient, was amplified via PCR and cloned into BamHI and XhoI sites of the same vector. Specific alanine mutations were inserted within the refp17 primary sequence by replacing the Trp at position 16, the Tyr at position 29, or both, generating the p17^W16A^, the p17^Y29A^, and the double-mutated p17^W16A+Y29A^, respectively, by using the Quick-Change Site-Directed Mutagenesis Kit (Agilent Technologies, Santa Clara, CA, USA). The recombinant proteins were further purified (>98%) via reverse-phase fast performance liquid chromatography (FPLC). The absence of LPS contamination (<0.25 endotoxin units/mL) in protein preparation was assessed via Limulus amoebocyte assay (Associates of Cape Cod, East Falmouth, MA, USA). The monoclonal antibody (mAb) to p17, named MBS-3, was produced and purified in our laboratory, as previously described [31,32].

### 2.7. B Cell Colony Formation Assay 

The assay was performed as previously described [13]. Briefly, Raji cells were seeded into a 96-well plate at a dilution of 0.5 cells/well. Plates were incubated for 8 days under standard conditions (RPMI medium supplemented with 10% FBS) in the presence or absence of 0.01 μg/mL of refp17, NHL-a101, p17^W16A^, p17^Y29A^, or p17^W16A+Y29A^ vp17s. Eight days after, culture plates were analyzed for single-colony formation. The colony area (15 colonies/condition) was measured using the Leica Qwin image analysis software (version 3.1.0). The same number of colonies (15 colonies/condition) was aseptically harvested from 96-well plates, and we stained them with propidium iodide (PI) to detect PI-viable cells via flow cytometry. Absolute cell counts were obtained by the counting function of the MACSQuant^®^ Analyzer (Miltenyi Biotec, Bergish Gladbach, Germany). When reported, refp17^W16A+Y29A^ was pre-incubated for 30 min at 37 °C with 1 μg/mL of unrelated control mAb (Ctrl mAb) or p17 neutralizing mAb MBS-3.

### 2.8. Statistical Analysis

Data were analyzed for statistical significance using the chi-square test for trend and evaluated with a confidence level (α) of 0.05. Data concerning biological assays were analyzed for statistical significance using one-way ANOVA. Bonferroni’s post hoc test was used to compare data. Differences were considered significant at *p* < 0.05. Statistical tests were performed using GraphPad Prism 8 software (GraphPad, San Diego, CA, USA).

## 3. Results

### 3.1. A Double Amino Acid Insertion Induces Changes in the Hydrophobic Profile of vp17s 

**Figure 1:**
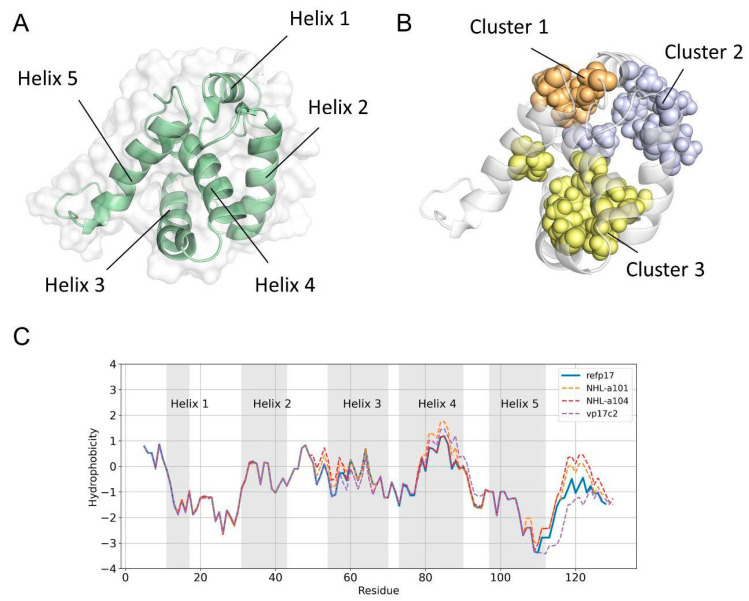
Hydrophobic profile and structural features of refp17 and vp17s. (**A**) The structure of refp17, showing the overall folding of the protein, is represented as a green cartoon and a white surface. (**B**) ILV clusters of refp17: cluster 1 in orange, cluster 2 in purple, and cluster 3 in yellow. The residues forming the clusters are shown as a Corey–Pauling–Koltun (CPK) model. (**C**) Comparison of the hydrophobic profile of refp17 and vp17s. Profiles were computed using the Kyte and Doolittle hydropathicity scale.

Differently from refp17, vp17s were found to trigger B cell growth and clonogenicity [7,9]. To understand if this gain of function could be related to alterations in the vp17s’ hydrophobic profile leading to conformational changes, hydrophobic interactions were assessed in refp17 by identifying so-called isoleucine, leucine, and valine (ILV) clusters, where the side chains of ILV form hydrophobic clusters (HCs) acting as the core of protein stability [33]. Analysis of refp17 identified three ILV clusters (Figure 1A,B). Subsequently, we performed the same analysis, taking into consideration the sequences of vp17s displaying, in addition to other aa mutations, distinct insertions, the first consisting of a glutamic acid (E) and a lysine (K) insertion at positions 114–115 (vp17c2) [9], and the second comprising two alanines (AA) at positions 117–118 (NHL-a101 and NHL-a104) [7]. Data obtained indicate that mutations in vp17s have no significant impact on the ILV clusters (Table S2). Then, we analyzed the hydrophobic profiles of refp17, taking into consideration both mutations and insertions. As shown in Figure 1C, refp17 exhibited a slight increase in hydrophobicity in the initial portion of H3, as compared to NHL-a101 and NHL-a104, and in the terminal portion of H4, as compared to NHL-a104 and vp17c2, thus confirming the low impact of mutations in vp17s. Interestingly, a substantial increase in the hydrophobic profile was observed in the C-terminus of NHL-a101 and NHL-a104, along with a strong decrease in vp17c2 caused by the double aa insertions (Figure 1C).

### 3.2. Refp17 and vp17s Show Critical Behavioral Differences 

**Figure 2:**
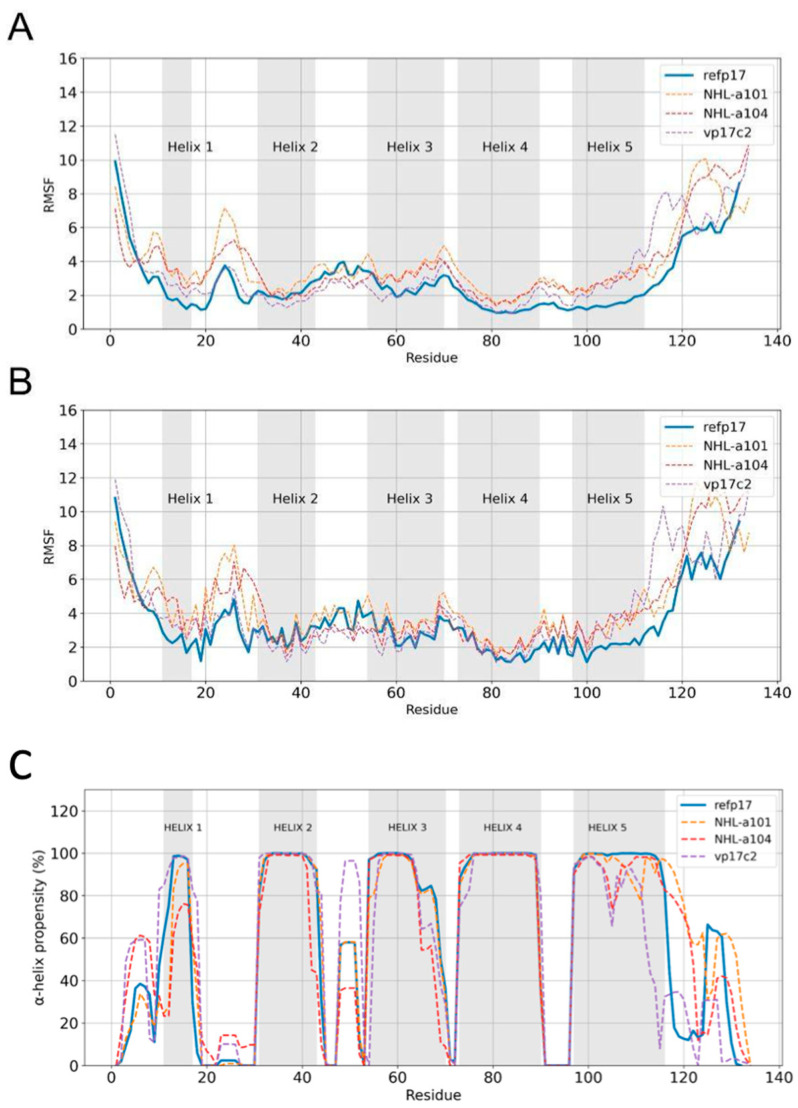
Protein residue fluctuations analysis and α-helix propensity comparison. RMSF calculation based on the (**A**) backbone of the proteins and on the (**B**) side chains. The gray regions correspond to the residues that form the α-helices. RMSF values of refp17 and vp17s are shown in solid and dashed lines, respectively. (**C**) Average of the α-helix propensity profile of refp17 and vp17s calculated from 300 ns of aMD. Values of refp17 and vp17s are shown with solid and dashed lines, respectively.

To establish and correlate the structural behavior of vp17s, accelerated aMDs were carried out in order to enhance conformational sampling during the simulation [28]. Comparative analysis of the molecular dynamics trajectories of refp17 and vp17s revealed critical differences. To better understand these differences, we quantified the fluctuation of every residue of each protein via evaluation of the RMSF values. Data obtained showed an overall increased flexibility in vp17s. Interestingly, the highest changes in flexibility were mainly found in the residues spanning between aa 8–32 of NHL-a101 and NHL-a104, the region in which the functional epitope for B cell clonogenicity is located [12]. A second area of changes in flexibility was highlighted in the C-terminal of the protein, specifically between residues 111 and 120, 121 and 130, and 121 and 132 in the vp17c2 (EK insertion), NHL-a101 (AA insertion), and NHL-a104 (AA insertion), respectively (Figure 2A,B). Taken together, these results revealed substantial differences between refp17 and vp17s.

Thus, to gain deeper insight into the cause of the high fluctuation of vp17s, we further studied and compared the proteins via secondary-structure elements analysis. As shown in Figure 2C, the final portion of H3 in NHL-a104 and vp17c2 and of H1 in NHL-a104 possess a lower helix propensity than refp17. Moreover, in refp17, the H5 extended from residues 97 to 116, with a stable α-helix becoming evident after 300 ns of aMD (Figure S2). Interestingly, all vp17s showed a destabilized H5 (Figure 2C). In NHL-a101, the formation of an initial short loop between residues 112 and 114 and a second loop between residues 107 and 111 is evident in the early (100–300 ns) and intermediate (500–600 ns) parts of the simulation, respectively. On the other hand, in the NHL-a104 variant, the formation of a short loop between residues 104 and 107 in the earliest part of the simulation (100–400 ns) and then a shortening of H5 up to residue 114 in the final 100 ns of the simulation was observed. Finally, in vp17c2, H5 was found to be unstable, extending to residue 103 only at the end of the simulation (Figure S2). Overall, the observed changes in the α-helix propensity can be related to the B cell clonogenic activity of vp17s. It is well known that changes in secondary structure can strongly impact the function of a protein, as the formation of some specific interactions is tied to the gain of function [34,35]. Moreover, it is intriguing to point out that the matrix protein (MA) of simian immunodeficiency virus (SIV) is known to possess B cell clonogenic activity [36]. It is worth noting that SIV MA possesses a truncated H5 as compared to the HIV-1 refp17, and that the C-terminal region of SIV p17 is known to fold in a β-sheet [37]. The instability of H5 in vp17s may have a functional correlation with the truncated H5 of the SIV MA.

### 3.3. Vp17s Loss of Interaction

To further study the different behavior between refp17 and vp17s, we carried out an H-bond and hydrophobic interactions analysis. H-bond analysis revealed that compared to refp17, the NHL-a101, NHL-a104, and vp17c2 vp17s lost 10, 9, and 12 interactions (<75% of lifetime), respectively (Table S3). 

**Figure 3:**
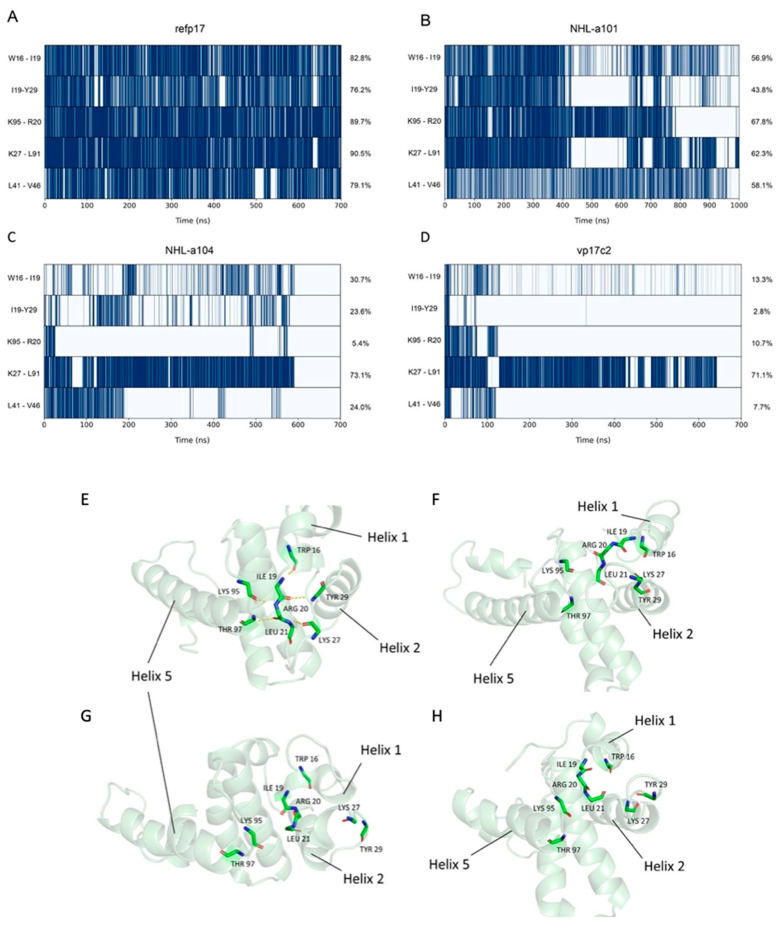
Hydrogen bond analysis. (**A**) H-bond occupancy as observed in refp17, (**B**) NHL-a101, (**C**) NHL-a104, and (**D**) vp17c2 during the aMD. *Y*-axis shows residues and atoms of each H-bond. Colors are set according to the presence (dark blue) or absence (light blue) of the interaction. (**E**–**H**) Residues involved in the H-bond network in refp17 and vp17s are shown. (**E**) A stable H-bond network in refp17 is observed along aMD. In contrast, in (**F**) NHL-a101, (**G**) NHL-a104, and (**H**) vp17c2, the H-bond network is disrupted.

Moreover, five of these loosened interactions were common between vp17s (Figure 3A–D), and four of them affected an H-bond network that, in refp17, is formed by Trp16, Ile19, Arg20, Leu21, Lys27, Tyr29, Lys95, and Thr97 (Figure 3E–H).

This network stabilized a mixed three-stranded β-sheet that plays a crucial role in establishing connections between H1, H2, and H5 and maintaining the tertiary structure of this refp17 region [38,39]. Interestingly, this H-bond network also involves residues from the N-terminal region of the protein, where the functional epitope responsible for B cell clonogenicity is located [12]. 

**Figure 4:**
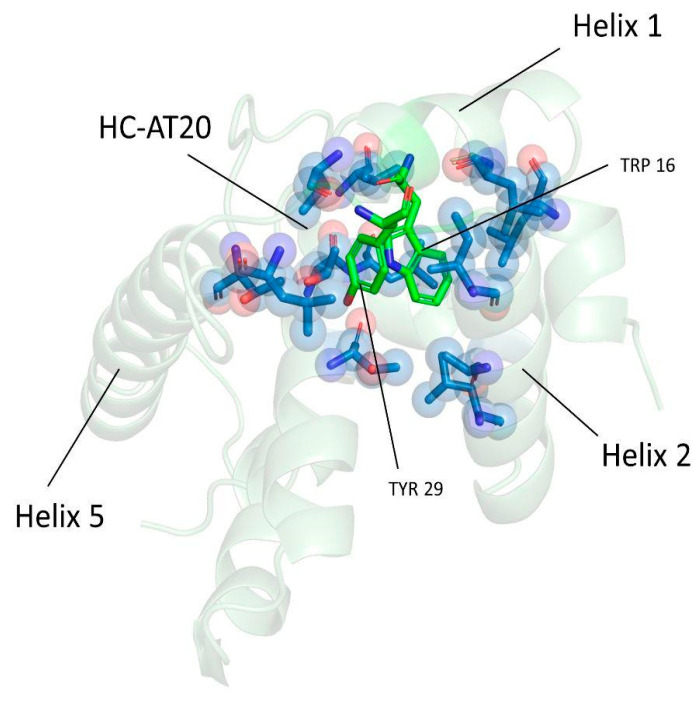
Representation of the hydrophobic cluster near the AT20 region (HC-AT20). The refp17 protein is represented as a cartoon; Trp16 and Tyr29 are denoted by green sticks, while the other residues forming the cluster in blue sticks.

Then, starting from the analysis of the hydrophobic interactions (Table S4), we focused our attention on those lost (<60%) near the region termed AT20, which includes the functional epitope, and in which the H-bond network described above was found to be disrupted in vp17s. Through visual inspection, we identified an HC (HC-AT20) formed by Leu8, Leu13, Trp16, Ile19, Leu21, Tyr29, Ile34, Ala37, Leu41, Thr81, Thr84, Leu85, Val88, and Thr97 (Figure 4). Trp16 and Tyr29 were found to be the core residues of the HC under investigation since the cluster is organized around them.

These results highlight a loss of interactions in a critical region of refp17. Moreover, Trp16 and Tyr29 were hypothesized to be key residues of the HC-AT20, possibly playing a crucial role in maintaining the stability of the protein globular domain.

### 3.4. Trp16 and Tyr29 Play a Key Role in Maintaining the Stability of HC-AT20 

**Figure 5:**
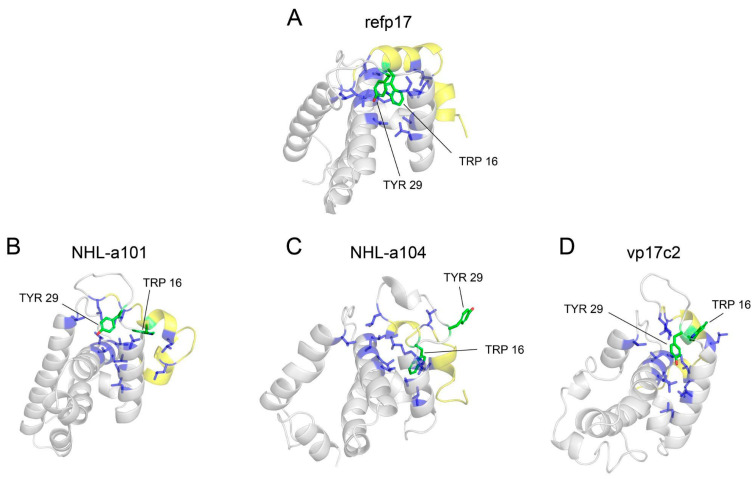
Disengagement of Trp16 and Tyr29 from HC-AT20 in B cell clonogenic vp17s. Starting from a stable HC-AT20 in refp17, the loss of H-bonds makes the region of the functional epitope AT20 (yellow cartoon) more flexible and unstable, ultimately leading to disruption of the HC-AT20 in all vp17s. (**A**) refp17; (**B**) NHL-a101; (**C**) NHL-a104; and (**D**) vp17c2. Trp16 and Tyr29 are indicated with green sticks, and HC-AT20 is indicated via a blue cartoon and sticks.

To investigate whether Trp16 and Tyr29 contribute to the refp17 globular conformation, we analyzed the behavior of these residues during aMD through dihedral angles analysis. In refp17, the chi2 values of Trp16 displayed variations from 0° to −100°. In contrast, the same angle exceeded 100° in vp17s (Figure S3). Similarly, chi1 values of Tyr29, which, in the refp17, were mainly close to −100°, underwent larger variations from −200° to 200° in vp17s (Figure S4). These data highlight the different side chain orientations of Trp16 and Tyr29 in vp17s during the simulations as compared to those in refp17. The different oscillation of the Trp16 and Tyr29 dihedral angles in vp17s was also confirmed by the study of vp17 structures extracted from aMD as these two residues were found to disengage from HC-AT20 in all B cell clonogenic vp17s, leading to the disruption of the HC under investigation (Figure 5).

Disruption of the HC-AT20 by Trp16 and Tyr29 disengagement further explains the conformational changes observed in vp17s, resulting in the induction of partial misfolding of refp17 near the AT20 region.

### 3.5. Conformational Changes in the AT20 Region of vp17s 

**Figure 6:**
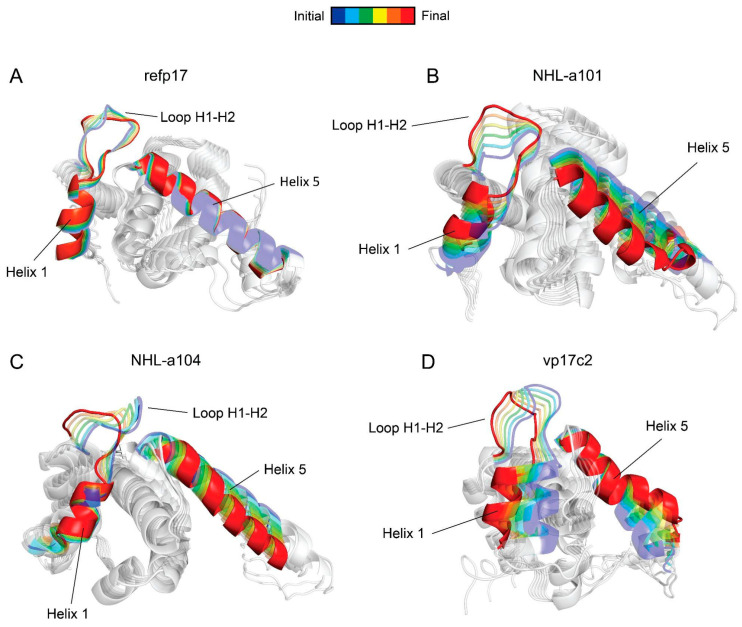
Motion across the first principal component. The viral proteins (**A**) refp17, (**B**) NHL-a101, (**C**) NHL-a104, and (**D**) vp17c2 are represented as cartoons. H1, H5, and the H1-H2 loop are highlighted in colors from the start (blue) to the end (red) of the motion.

We applied PCA to examine the conformational motions that mainly represent the dynamics of refp17 and vp17s. PCA is a widely employed method for studying the collective dynamics of proteins, and it is of primary interest when investigating large conformational changes [40]. As shown in Figure 6A, refp17 showed a stable H5, with no significant movement of the N-terminal portion of the protein, where the active epitope is located. Conversely, all vp17s displayed a loss of compactness in the loop between H1 and H2 (Figure 6B–D), which is less constrained to the globular portion of the protein. Furthermore, the movement of H1 and the instability of H5 in NHL-a101 and vp17c2 were also evident. This result highlights the conformational changes occurring in vp17s as compared to refp17 and allows us to hypothesize that the AT20 epitope of vp17s may likely be more exposed and suitable for fitting with PAR-1 than the one in the refp17.

### 3.6. Trp16 and Tyr29 Are Fundamental Core Residues for the Structural Maintenance of the HC-AT20

To investigate the importance of Trp16 and Tyr29 in stabilizing the HC-AT20, an in silico alanine scanning approach was employed. Trp16 and Tyr29 were mutated to alanine in refp17, and the obtained refp17^W16A+Y29A^ 3D model was subjected to aMD, as previously described. Trajectory analysis revealed no significant increases in fluctuations of the refp17^W16A+Y29A^ in comparison to refp17 (Figure S5). However, H5 suffered a total loss of secondary structure in the region corresponding to residues 107 to 109. This led to a segmentation of the H5, similar to what was observed in vp17s (Figure S6). Moreover, the same common H-bonds lost in vp17s were missing in refp17^W16A+Y29A^, as well as the loss of hydrophobic interactions and disruption of the HC-AT20 due to the absence of Trp16 and Tyr29 (Figure S7 and Table S5). Overall, this result proves the pivotal role played by Trp16 and Tyr29 in maintaining the refp17 globular structure near the AT20 domain. Ultimately, it is also conceivable that refp17^W16A+Y29A^ may acquire a B cell clonogenic phenotype similar to that of NHL-a101, NHL-a104, and vp17c2 vp17s.

### 3.7. Replacement of Trp16 and Tyr29 with Alanine on the refp17 Backbone Confers B Cell Clonogenic Activity to the Viral Protein

**Figure 7:**
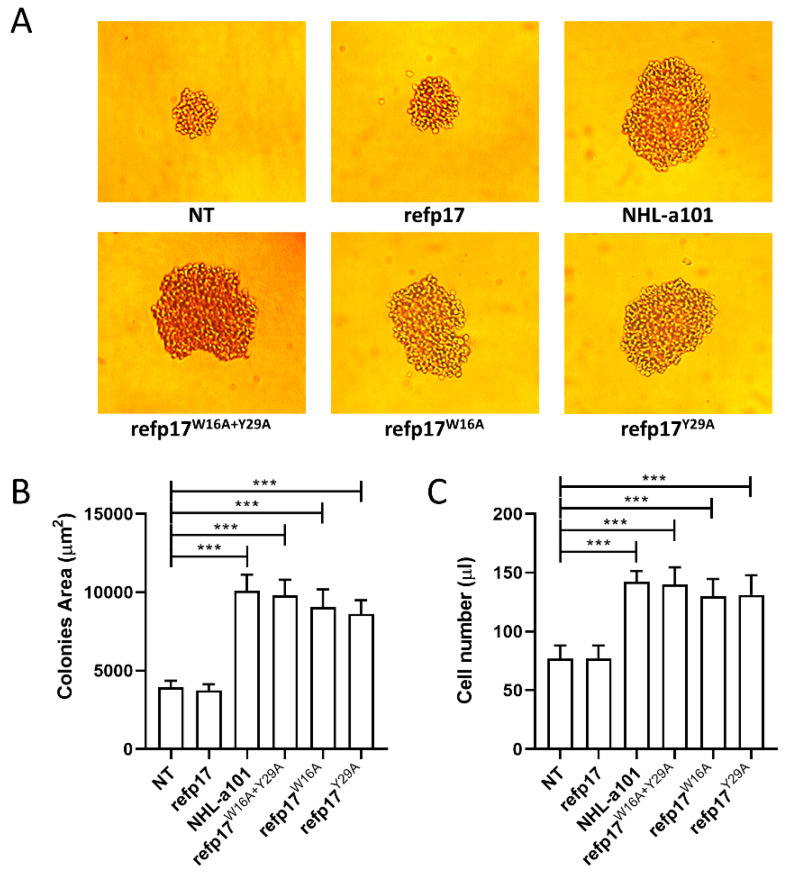
Effect of refp17^W16A+Y29A^, refp17^W16A^, and refp17^Y29A^ on B cell growth and clonogenicity. In the single-cell colony formation assay, Raji cells were cultured for 8 days in the presence or absence of refp17, NHL-a101, refp17^W16A+Y29A^, refp17^W16A^, or rep17^Y29A^ (0.01 μg/mL). (**A**) Bright-field images represent the characteristic morphology of two-dimensional colonies (original magnification: ×40). (**B**) The colony area was measured using Leica Qwin image analysis software. (**C**) The same number of colonies were aseptically harvested from 96-well plates and stained with PI to detect viable cells via flow cytometry. Absolute cell counts were obtained by the counting function of the MACSQuant Analyzer. Bars represent the means ± SD of three independent experiments. The statistical significance was calculated using one-way ANOVA, and Bonferroni’s post hoc test was used to compare data. NT, not treated cells. *** p < 0.001.

Based on evidence obtained by the MDs, a proof-of-concept experiment was performed to define, definitively, the crucial role played by Trp16 and Tyr29 in preserving the stability and the exposure of the B cell clonogenic epitope within the AT20 domain. We engineered the inactive refp17 by replacing Trp16 and Tyr29, alone or in combination, with Ala, and the mutated refp17s were evaluated for their capability to enhance the clonogenic activity of Raji lymphoma B cells. With this aim, a single-cell cloning assay was carried out [11]. This method was performed by seeding 0.5 B cells in each well of a 96-well plate in the presence or absence of p17^W16A+Y29^ (bearing Trp16 and Tyr29 mutations), p17^W16A^ (bearing Trp16 mutation), p17^Y29A^ (bearing Tyr29 mutation), or the clonogenic NHL-a101 at a concentration of 0.01 μg/mL [13]. At day 8 of culture, Raji cells formed a visible single colony in >60% of seeded wells, attesting to active cell proliferation. As shown in Figure 7A,B, refp17^W16A+Y29^, refp17^W16A^, and refp17^Y29A^, differently from their natural counterpart refp17, promoted the formation of colonies with a drastically larger size as compared to cells cultured in medium alone (not treated, NT). A similar result was obtained with the NHL-a101 vp17 used as a positive control in the single-cell clonogenic assay [7]. A cell suspension was obtained by pooling equal numbers of colonies per each experimental condition, and the absolute number of cells was evaluated via PI staining and flow cytometry. As shown in Figure 7C, refp17^W16A+Y29A^, refp17^W16A^, refp17^Y29A^, or NHL-a101 significantly increased B cell proliferation as compared with cells treated with refp17 or NT. 

### 3.8. The p17-Neutralizing mAb MBS-3 Impairs the refp17^W16A+Y29A^ Cell-Growth-Promoting Activity

**Figure 8:**
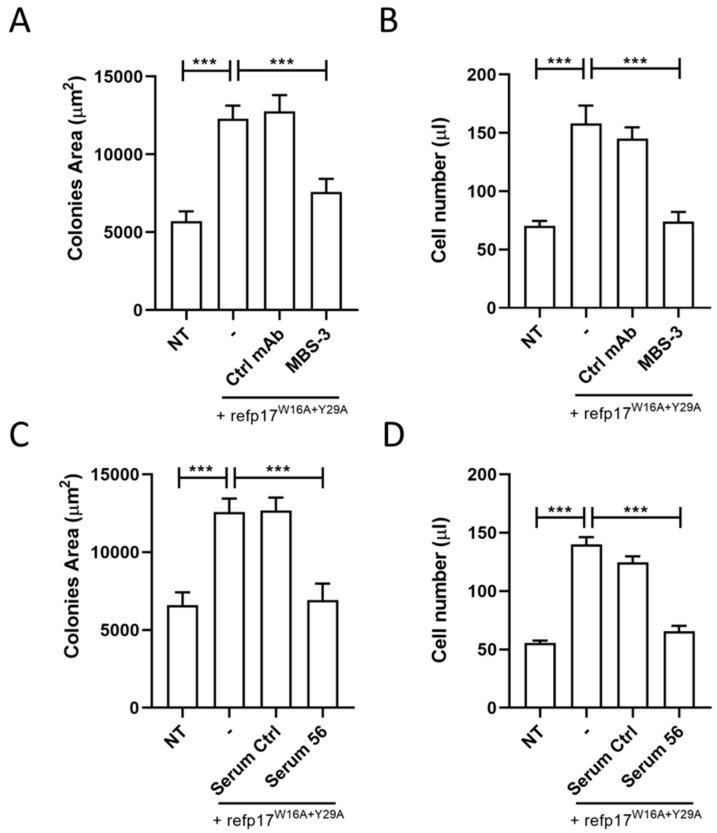
Neutralization of refp17^W16A+Y29A^ clonogenicity using the anti-p17 mAb MBS-3 and a serum derived from an HIV^+^ patient immunized with AT20-KLH. In the colony formation assay, Raji cells were cultured for 8 days in the presence or absence of refp17^W16A+Y29A^ (0.01 μg/mL). For some experimental conditions, the viral protein was pre-incubated for 30 min with 1 μg/mL of mAb MBS-3 or of a Ctrl (**A**,**B**), or with a 1:10 dilution of serum 56 or of serum Ctrl (**C**,**D**). (**A**,**C**) The colony area was measured using Leica Qwin image analysis software. (**B**,**D**) The same number of colonies were aseptically harvested from 96-well plates and stained with PI to detect viable cells via flow cytometry. Absolute cell counts were obtained by the counting function of the MACSQuant Analyzer. Bars represent the means ± SD of three independent experiments performed in triplicate. The statistical significance was calculated using one-way ANOVA, and Bonferroni’s post hoc test was used to compare data. NT, not treated cells. *** p < 0.001.

In order to confirm that the gain of B cell clonogenic function showed by refp17 arises from the exposure of the AT20 epitope, following the W16A and Y29A double mutations, as predicted by in silico data, we assess the capability of specific p17 mAb to neutralize the clonogenic activity of the refp17^W16A+Y29A^. To this end, we performed a single-cell cloning assay in the presence or absence of an unrelated mAb (Ctrl) or of the p17 neutralizing mAb, named MBS-3, which recognizes the AT20 epitope of refp17, included between aa 9 and 22 [31]. As expected, in the presence of 0.01 μg/mL of refp17^W16A+Y29A^, colonies showed a significantly *(p* < 0.001) larger size than colonies formed by NT cells. Interestingly, the presence of the mAb MBS-3 (1 μg/mL) completely inhibited the clonogenic activity of refp17^W16A+Y29A^, whereas the Ctrl mAb (1 μg/mL) proved to be ineffective (Figure 8A). As shown in Figure 8B, the refp17^W16A+Y29A^-triggered cell proliferation was also significantly inhibited by the presence of the mAb MBS-3 but not by the presence of the Ctrl mAb. These results demonstrate that the clonogenic activity induced by Trp16 and Tyr29 mutations can be neutralized by the specific MBS-3 mAb, targeting the N-terminal region of p17, thus indicating that those aa residues are not affecting its neutralizing activity.

### 3.9. Serum Derived from an HIV^+^ Patient Immunized with AT20-KLH Suppressed the refp17^W16A+Y29A^ Clonogenic Activity

During the typical course of HIV-1 infection, Ab response to the p17 functional region is rare [5]. Indeed, serum from HIV^+^ patients was discovered to be unable to neutralize the biological activity of the viral protein despite the presence of high-titer anti-p17 abs [41]. Interestingly, during a therapeutic phase-I clinical trial, HIV^+^ patients immunized with a 20-aa-long synthetic peptide, named AT20, representative of the p17 functional region, developed high titers of high-avidity p17 neutralizing Abs [42]. Here, we evaluated the efficacy of a serum, derived from an HIV^+^ patient immunized with AT20-KLH, in inhibiting the refp17^W16A+Y29A^ clonogenic activity. We performed a single-cell cloning assay by pre-incubating the refp17^W16A+Y29A^ for 30 min at 37 °C with a 1:10 dilution of the selected serum, collected 56 days post-immunization (serum 56; anti-AT20 Abs titer > 1000). As a control, we incubated the viral protein with a serum (serum Ctrl) derived from the same patient but collected before immunization (anti-AT20 Abs titer < 10). As shown in Figure 8, serum 56 was able to significantly reduce the size of the colonies (panel C) and completely inhibit the B cell proliferation (panel D) induced by refp17^W16A+Y29A^ as compared to NT. On the other hand, as expected, serum Ctrl was found to be unable to neutralize the growth-promoting effects of refp17^W16A+Y29A^ on B cells (Figure 8C,D). These results suggest that vaccination with the AT20-KLH is able to redirect the Ab response against a specific epitope by hampering the interaction between vp17s and their receptor, thereby blocking the vp17s clonogenic activity on B cells.

## 4. Discussion

Extracellularly, refp17 was found to deregulate the biological activity of relevant cell populations in the context of HIV-1 pathogenesis [5]. Differently from refp17, vp17s, more frequently detected in the plasma of HIV-1^+^ patients with rather than without lymphoma, were found to be capable of triggering B cell growth and clonogenicity [7,9]. In particular, this activity is exerted by peculiar categories of vp17s characterized by aa insertions always confined in the C-terminal region of the viral protein, specifically at positions 114–115, 117–118, and/or 125–126 [7,9]. In a properly folded state, refp17 does not exert any biological activity on B cell growth and clonogenicity. Nevertheless, it is worth noting that changes in the secondary-structure element can strongly impact the function of a protein [34,35]; indeed, vp17s endowed with B cell growth-promoting activity are drastically destabilized [7,8]. Earlier, we demonstrated that the insertion of two Ala residues at position 117–118 [7], or a single Arg to Gly mutation at position 76 [12], or forcing a disulphide bridge between two Cys residues at position 57 and 87 [13] in refp17 results in misfolded proteins that display a potent B cell clonogenic activity. Moreover, we revealed that the presence of H5 in refp17 is not necessary for protein clonogenicity, highlighting the intrinsic capability of refp17 to exert B-cell-growth-promoting activity following protein unfolding [8]. In fact, truncation of the last 36 aa residues of refp17 resulted in a protein (p17Δ36) capable of promoting B cell clonogenicity. H-bonds provide most of the directional interactions that underpin protein folding [43]. Thus, it is plausible that the gain of function of vp17s could be ascribed to conformational changes due to modification in the secondary-structure elements and in the rearrangement of the H-bond network, which induces the exposure of a camouflaged functional region. Previous computational studies have suggested how a single aa substitution in refp17 is able to induce strong conformational changes due to the breakdown of the H-bond network, causing destabilization and disorganization of the viral protein [12]. This finding led us to hypothesize that a specific epitope, located in the N-terminal region of the protein and responsible for B cell clonogenic activity, is masked in the properly folded refp17 but exposed in misfolded vp17s [13]. Therefore, the molecular reason for the opposite mechanisms evident among mutated proteins and refp17 is likely to depend on the exposure (or not) of the clonogenic epitope. Previous data, obtained by dissecting refp17, allowed us to recognize the masked functional epitope endowed with B cell clonogenic activity, which is located at the N-terminal region of the viral protein overlapping the AT20 region, a previously identified loop representing a hot spot of biological activity [13]. We also highlighted the critical role played in B cell clonogenicity by three positively charged aa, namely, Arg15, Lys18, and Arg20. Therefore, it is more likely that those critical aa residues shared by refp17 and vp17s are more exposed in the latter. 

The clonogenic functional epitope of p17 is surrounded by hydrophobic aa [13]. It is known that hydrophobic cores are fundamental structural assets of proteins which are classically correlated with their folding and stability. Nevertheless, they characterize protein evolution and function, even if how this occurs is not yet fully understood [44]. As for refp17, we have solved this enigma. Here we demonstrate that a single aa mutation in the p17 globular domain, located in the N-terminal region of the viral protein, switches an inactive refp17 protein to a biologically active one able to promote B cell clonogenicity. This was computationally found to occur through the induction of conformational changes, resulting in a partial misfolding of refp17 near the AT20 region, which increases the flexibility and exposure of functional residues, altering and destabilizing the structure of the viral protein. Specifically, destabilization of vp17s occurred following the disruption of a specific hydrophobic cluster, termed HC-AT20, whose stabilization is linked to Trp16 and Tyr29. Interestingly, we found that in all vp17s, these residues disengaged from the cluster, leading to conformational changes and rendering the functional epitope more accessible for binding to the receptor PAR-1. Eventually, an in silico alanine scanning approach confirmed the pivotal role of Trp16 and Tyr29 in stabilizing HC-AT20. Indeed, virtual mutations of these residues in the refp17 backbone resulted in structural changes resembling those observed in vp17s, suggesting their fundamental importance in maintaining the globular structure and the functionality of the protein. 

The hypothesis raised on the basis of data obtained through computational data modeling was confirmed by biological studies. The introduction of the two aa mutations in refp17, forcing the disruption of the HC-AT20, more likely resulted in partially misfolded proteins displaying potent B cell clonogenic activity. Indeed, proof-of-concept experiments revealed that mutation of Trp16 and Tyr29, alone or in combination, with Ala, which possesses a very small side chain, led to the generation of three mutated refp17s, namely, refp17^W16A^, refp17^Y29A^, and refp17^W16A+Y29^, endowed with B cell clonogenic activity. It is worth noting that p17 is produced as a myristoylated (myr) protein. The myristoyl group is known to stabilize the HIV-1 matrix protein [45] and to interact with residues forming the HC-AT20 [46]. Previously, we demonstrated that myr vp17s—but not myr refp17—released from HIV-1 Gag-expressing cells induce B cell growth and clonogenicity [47]. Based on this evidence, myristoylation does not affect the clonogenic activity of vp17s; therefore, in silico analyses were here conducted on non-myristoylated proteins. 

Vp17s were recently described as critical microenvironmental factors promoting lymphoma development, changing the old paradigm which assumed that HIV-1 is only indirectly related to lymphomagenesis [7,8,9,10,11]. Both refp17 and vp17s are secreted in a biologically active form in the extracellular microenvironment by an unconventional pathway [4,47]. Their secretion takes place at the plasma membrane in the absence of active viral proteases and is dependent on the interaction of Gag precursor polyprotein (Pr55Gag) with phosphatidylinositol-(4,5)-bisphosphate (PI(4,5)P2) and its subsequent cleavage from Pr55Gag operated via cellular aspartyl proteases [4,47]. These findings suggest that vp17s can also be released during HIV-1 latency, thus generating a microenvironment that fosters lymphoma development, progression, and metastasis.

Recent data have demonstrated that the prevalence of HIV-1 mutants expressing vp17s has been increasing, worldwide, over time [9]. More interestingly, we described a cluster of HIV-1 mutants expressing a B cell clonogenic vp17 and highlighted that insertions can be fixed and that viruses displaying clonogenic vp17s are actively spreading [9]. This is not surprising since it is worth noting that mutations in the C-terminal region of refp17 do not affect viral replication and infectivity [48], and virions carrying vp17s do assemble correctly [47]. 

Previously, we demonstrated that despite their B cell clonogenic activity, vp17s are also able to promote angiogenesis [32] and lymphangiogenesis [49], equally supporting lymphoma proliferation and tumor cell dissemination [50]. These functional activities were found to occur after the interaction between a functional epitope located within the AT20 loop with two G-coupled receptors expressed on human endothelial cells, namely, CXCR-1 and CXCR-2 [49]. Altogether, these data highlight the capability of vp17s to drive lymphomagenesis through a “two compartments” activity and pave the way for the implementation of new therapeutic approaches for the prevention or treatment of lymphoma in the context of HIV-1 infection. 

The functional epitopes responsible for B cells clonogenicity, angiogenesis, and lymphangiogenesis reside within a domain targeted by the synthetic HIV-1 p17-based AT20-KLH therapeutic vaccine [51]. We showed that AT20-KLH vaccination of highly active antiretroviral therapy (HAART)-treated patients redirected a long-lasting and neutralizing humoral response toward the previously untargeted hotspot of functional activity represented by the AT20 loop [41,42]. Here, we show that antibodies against the AT20 epitope, evoked by AT20-KLH vaccination, are able to neutralize the clonogenic activity endowed by refp17^W16A+Y29A^. Interestingly, the same antibodies were found to neutralize the capability of viral proteins to promote angiogenesis and lymphangiogenesis [32,47,49]. Recognizing the interaction of B cell clonogenic vp17s with PAR-1, CXCR1, and CXCR2 as key events in sustaining lymphoma growth and dissemination prompts us to study whether the AT20-KLH therapeutic vaccine may represent a promising strategy for fighting AIDS-related lymphomas. Additionally, the combination of MDs, artificial intelligence (AI), and machine learning (ML) will be helpful in predicting novel and/or repurposing existing drugs that may be effective in targeting the AT20 region, as well as in proposing new strategies for providing anticancer therapeutic benefits not only in terms of antiproliferative lymphoma B cells but also in terms of inhibiting the neovascularization process needed for cancer growth and metastatization. 

In conclusion, our study clarifies the structure–function relationship causing B cell growth and clonogenicity in vp17s carrying peculiar aa insertions in their C-terminal region and identifies a novel potential therapeutic target to counteract vp17-driven lymphomagenesis in HIV-1^+^ patients.

## Data Availability

All study data are included in the article and/or Supporting Information.

## Supplementary Materials

The following supporting information can be downloaded at https://www.mdpi.com/article/10.3390/v16071048/s1: Table S1: Model systems simulated with aMD; Figure S1: Protein dihedral angles; Table S2: Mapping hydrophobic clusters; Figure S2: Time series of α-helix propensity; Table S3: Hydrogen bond frequencies of refp17 and vp17s during aMD; Table S4: Hydrophobic interactions analysis; Figure S3: Comparative dihedral angle distribution analysis; Figure S4: Comparative dihedral angle distribution analysis; Figure S5: Protein residue fluctuations analysis; Figure S6: α-helix propensity comparison; Figure S7: Hydrogen bond time series; Table S5: Hydrophobic interactions analysis.

## References

[1] Fiorentini S., Marini E., Caracciolo S., Caruso A. (2006). Functions of the HIV-1 matrix protein p17. New Microbiol..

[2] Mazzuca P., Caruso A., Caccuri F. (2016). HIV-1 infection, microenvironment and endothelial cell dysfunction. New Microbiol..

[3] Popovic M., Tenner-Racz K., Pelser C., Stellbrink H.J., van Lunzen J., Lewis G., Kalyanaraman V.S., Gallo R.C., Racz P. (2005). Persistence of HIV-1 structural proteins and glycoproteins in lymph nodes of patients under highly active antiretroviral therapy. Proc. Natl. Acad. Sci. USA.

[4] Caccuri F., Iaria M.L., Campilongo F., Varney K., Rossi A., Mitola S., Schiarea S., Bugatti A., Mazzuca P., Giagulli C. (2016). Cellular aspartyl proteases promote the unconventional secretion of biologically active HIV-1 matrix protein p17. Sci. Rep..

[5] Fiorentini S., Giagulli C., Caccuri F., Magiera A.K., Caruso A. (2010). HIV-1 matrix protein p17: A candidate antigen for therapeutic vaccines against AIDS. Pharmacol. Ther..

[6] Hill C.P., Worthylake D., Bancroft D.P., Christensen A.M., Sundquist W.I. (1996). Crystal structures of the trimeric human immunodeficiency virus type 1 matrix protein: Implications for membrane association and assembly. Proc. Natl. Acad. Sci. USA.

[7] Dolcetti R., Giagulli C., He W., Selleri M., Caccuri F., Eyzaguirre L.M., Mazzuca P., Corbellini S., Campilongo F., Marsico S. (2015). Role of HIV-1 matrix protein p17 variants in lymphoma pathogenesis. Proc. Natl. Acad. Sci. USA.

[8] Giagulli C., Marsico S., Magiera A.K., Bruno R., Caccuri F., Barone I., Fiorentini S., Andò S., Caruso A. (2011). Opposite effects of HIV-1 p17 variants on PTEN activation and cell growth in B cells. PLoS ONE.

[9] Caccuri F., Messali S., Zani A., Campisi G., Giovanetti M., Zanussi S., Vaccher E., Fabris S., Bugatti A., Focà E. (2022). HIV-1 mutants expressing B cell clonogenic matrix protein p17 variants are increasing their prevalence worldwide. Proc. Natl. Acad. Sci. USA.

[10] Dolcetti R., Gloghini A., Caruso A., Carbone A. (2016). A lymphomagenic role for HIV beyond immune suppression?. Blood.

[11] Giagulli C., Caccuri F., Zorzan S., Bugatti A., Zani A., Filippini F., Manocha E., D’Ursi P., Orro A., Dolcetti R. (2021). B-cell clonogenic activity of HIV-1 p17 variants is driven by PAR1-mediated EGF transactivation. Cancer Gene Ther..

[12] Giagulli C., D’Ursi P., He W., Zorzan S., Caccuri F., Varney K., Orro A., Marsico S., Otjacques B., Laudanna C. (2017). A single amino acid substitution confers B-cell clonogenic activity to the HIV-1 matrix protein p17. Sci. Rep..

[13] He W., Mazzuca P., Yuan W., Varney K., Bugatti A., Cagnotto A., Giagulli C., Rusnati M., Marsico S., Diomede L. (2019). Identification of amino acid residues critical for the B cell growth-promoting activity of HIV-1 matrix protein p17 variants. Biochim. Biophys. Acta Gen. Subj..

[14] Miotto M., Olimpieri P.P., Di Rienzo L., Ambrosetti F., Corsi P., Lepore R., Tartaglia G.G., Milanetti E. (2019). Insights on protein thermal stability: A graph representation of molecular interactions. Bioinformatics.

[15] Priyakumar U.D. (2012). Role of hydrophobic core on the thermal stability of proteins—Molecular dynamics simulations on a single point mutant of Sso7d abstract. J. Biomol. Struct. Dyn..

[16] Van den Burg B., Dijkstra B.W., Vriend G., Van der Vinne B., Venema G., Eijsink V.G. (1994). Protein stabilization by hydrophobic interactions at the surface. Eur. J. Biochem..

[17] Banach M., Fabian P., Stapor K., Konieczny L., Roterman A.I. (2020). Structure of the Hydrophobic Core Determines the 3D Protein Structure-Verification by Single Mutation Proteins. Biomolecules.

[18] Fabian P., Stapor K., Banach M., Ptak-Kaczor M., Konieczny L., Roterman I. (2020). Alternative Hydrophobic Core in Proteins—The Effect of Specific Synergy. Symmetry.

[19] Berman H.M., Westbrook J., Feng Z., Gilliland G., Bhat T.N., Weissig H., Shindyalov I.N., Bourne P.E. (2000). The Protein Data Bank. Nucleic Acids Res..

[20] Sali A.A., Blundell T.L. (1993). Comparative protein modelling by satisfaction of spatial restraints. J. Mol. Biol..

[21] Laskowski R.A., MacArthur M.W., Moss D.S., Thornton J.M. (1993). PROCHECK—A program to check the stereochemical quality of protein structures. J. App. Cryst..

[22] Studer G., Rempfer C., Waterhouse A.M., Gumienny R., Haas J., Schwede T. (2020). QMEANDisCo-distance constraints applied on model quality estimation. Bioinformatics.

[23] Case D.A., Ben-Shalom I.Y., Brozell S.R., Cerutti D.S., Cheatham III T.E., Cruzeiro V.W.D., Darden T.A., Duke R.E., Ghoreishi D., Gilson M.K. (2018). AMBER2018.

[24] Maier J.A., Martinez C., Kasavajhala K., Wickstrom L., Hauser K.E., Simmerling C. (2015). ff14SB: Improving the Accuracy of Protein Side Chain and Backbone Parameters from ff99SB. J. Chem. Theory Comput..

[25] Machado M.R., Pantano S. (2020). Split the Charge Difference in Two! A Rule of Thumb for Adding Proper Amounts of Ions in MD Simulations. J. Chem. Theory Comput..

[26] Hamelberg D., Mongan J., McCammon J.A. (2004). Accelerated molecular dynamics: A promising and efficient simulation method for biomolecules. J. Chem. Phys..

[27] Miao Y., Feixas F., Eun C., McCammon J.A. (2015). Accelerated molecular dynamics simulations of protein folding. J. Comput. Chem..

[28] Pierce L.C., Salomon-Ferrer R., Augusto F de Oliveira C., McCammon J.A., Walker R.C. (2012). Routine Access to Millisecond Time Scale Events with Accelerated Molecular Dynamics. J. Chem. Theory Comput..

[29] Durrant J.D., McCammon J.A. (2011). HBonanza: A computer algorithm for molecular-dynamics-trajectory hydrogen-bond analysis. J. Mol. Graph. Model..

[30] Tina K.G., Bhadra R., Srinivasan N. (2007). PIC: Protein Interactions Calculator. Nucleic Acids Res..

[31] De Francesco M.A., Baronio M., Fiorentini S., Signorini C., Bonfanti C., Poiesi C., Popovic M., Grassi M., Garrafa E., Bozzo L. (2002). HIV-1 matrix protein p17 increases the production of proinflammatory cytokines and counteracts IL-4 activity by binding to a cellular receptor. Proc. Natl. Acad. Sci. USA.

[32] Caccuri F., Giagulli C., Bugatti A., Benetti A., Alessandri G., Ribatti D., Marsico S., Apostoli P., Slevin M.A., Rusnati M. (2012). HIV-1 matrix protein p17 promotes angiogenesis via chemokine receptors CXCR1 and CXCR2. Proc. Natl. Acad. Sci. USA.

[33] Kathuria S.V., Chan Y.H., Nobrega R.P., Özen A., Matthews C.R. (2016). Clusters of isoleucine, leucine, and valine side chains define cores of stability in high-energy states of globular proteins: Sequence determinants of structure and stability. Protein Sci..

[34] Yue P., Li Z., Moult J. (2005). Loss of protein structure stability as a major causative factor in monogenic disease. J. Mol. Biol..

[35] Yue P., Melamud E., Moult J. (2006). SNPs3D: Candidate gene and SNP selection for association studies. BMC Bioinform..

[36] Feichtinger H., Putkonen P., Parravicini C., Li S.L., Kaaya E.E., Böttiger D., Biberfeld G., Biberfeld P. (1990). Malignant lymphomas in cynomolgus monkeys infected with simian immunodeficiency virus. Am. J. Pathol..

[37] Rao Z., Belyaev A.S., Fry E., Roy P., Jones I.M., Stuart D.I. (1995). Crystal structure of SIV matrix antigen and implications for virus assembly. Nature.

[38] Massiah M.A., Starich M.R., Paschall C., Summers M.F., Christensen A.M., Sundquist W.I. (1994). Three-dimensional structure of the human immunodeficiency virus type 1 matrix protein. J. Mol. Biol..

[39] Matthews S., Barlow P., Clark N., Kingsman S., Kingsman A., Campbell I. (1995). Refined solution structure of p17, the HIV matrix protein. Biochem. Soc. Trans..

[40] Galindo-Murillo R., Roe D.R., Cheatham T.E. (2015). Convergence and reproducibility in molecular dynamics simulations of the DNA duplex d(GCACGAACGAACGAACGC). Biochim. Biophys. Acta.

[41] Iaria M.L., Fiorentini S., Focà E., Zicari S., Giagulli C., Caccuri F., Francisci D., Di Perri G., Castelli F., Baldelli F. (2014). Synthetic HIV-1 matrix protein p17-based AT20-KLH therapeutic immunization in HIV-1-infected patients receiving antiretroviral treatment: A phase I safety and immunogenicity study. Vaccine.

[42] Focà E., Iaria M.L., Caccuri F., Fiorentini S., Motta D., Giagulli C., Castelli F., Caruso A. (2015). Long-lasting humoral immune response induced in HIV-1-infected patients by a synthetic peptide (AT20) derived from the HIV-1 matrix protein p17 functional epitope. HIV Clin. Trials.

[43] Hoang T.X., Trovato A., Seno F., Banavar J.R., Maritan A. (2004). Geometry and symmetry presculpt the free-energy landscape of proteins. Proc. Natl. Acad. Sci. USA.

[44] Venkat A., Tehrani D., Taujale R., Yeung W., Gravel N., Moremen K.W., Kannan N. (2022). Modularity of the hydrophobic core and evolution of functional diversity in fold A glycosyltransferases. J. Biol. Chem..

[45] Wu Z., Alexandratos J., Ericksen B., Lubkowski J., Gallo R.C., Lu W. (2004). Total chemical synthesis of N-myristoylated HIV-1 matrix protein p17: Structural and mechanistic implications of p17 myristoylation. Proc. Natl. Acad. Sci. USA.

[46] Saad J.S., Miller J., Tai J., Kim A., Ghanam R.H., Summers M.F. (2006). Structural basis for targeting HIV-1 Gag proteins to the plasma membrane for virus assembly. Proc. Natl. Acad. Sci. USA.

[47] Bugatti A., Caccuri F., Filippini F., Ravelli C., Caruso A. (2021). Binding to PI(4,5)P_2_ is indispensable for secretion of B-cell clonogenic HIV-1 matrix protein p17 variants. J. Biol. Chem..

[48] Bhatia A.K., Campbell N., Panganiban A., Ratner L. (2007). Characterization of replication defects induced by mutations in the basic domain and C terminus of HIV-1 matrix. Virology.

[49] Caccuri F., Rueckert C., Giagulli C., Schulze K., Basta D., Zicari S., Marsico S., Cervi E., Fiorentini S.M., Slevin C.A. (2014). HIV-1 matrix protein p17 promotes lymphangiogenesis and activates the endothelin-1/endothelin B receptor axis. Arterioscler. Thromb. Vasc. Biol..

[50] Paduch R. (2016). The role of lymphangiogenesis and angiogenesis in tumor metastasis. Cell. Oncol..

[51] Fiorentini S., Marini E., Bozzo L., Trainini L., Saadoune L., Avolio M., Pontillo A., Bonfanti C., Sarmientos P., Caruso A. (2004). Preclinical studies on immunogenicity of the HIV-1 p17-based synthetic peptide AT20-KLH. Biopolymers.

