# Supplementary material for: Molecular Mechanisms Involved in the B Cell Growth and Clonogenic Activity of HIV-1 Matrix Protein p17 Variants
Source: Viruses. 2024 Jun 28;16(7):1048. doi: 10.3390/v16071048 (PMC11281387; doi:10.3390/v16071048)
Supplement: Supplementary file 1 [file viruses-16-01048-s001.zip › viruses-3027507-supplementary.pdf]

Supplementary materials

System	N_{res}	N_{atoms}	cMD	aMD	Boost	Ed (kcal/mol)	αD (kcal/mol)	Ep (kcal/mol)	αP (kcal/mol)
refp17	132	64717	100 ns	700 ns	dual-boost	2099	92.4	-195907	12943
NHL-a101	134	52856	100 ns	1000 ns	dual-boost	2130	93.8	-159308	10571
NHL-a104	134	45108	100 ns	700 ns	dual-boost	2126	93.8	-135564	9022
vp17c2	134	52735	100 ns	700 ns	dual-boost	2159	93.8	-158800	10547
refp17$^{W16A+Y29A}$	132	49936	100 ns	700 ns	dual-boost	2095	92.4	-150684	9987

Supplementary Table S1. Model Systems simulated with aMD; N_{res} and N_{atoms} are the number of residues and the number of atoms in the system, respectively. Ed, αD, Ep, αP are the average dihedral energy threshold, the inverse strength boost factor for the dihedral energy, the average total potential energy threshold, and the inverse strength boost factor for the total potential energy, respectively.

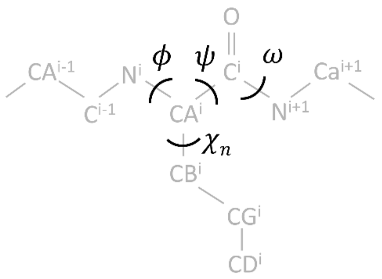

Supplementary Figure S1. Protein dihedral angles: for the protein backbone, Φ (phi) is defined by the sequence C^{i-1}-N^i-CA^i-C^i, Ψ (psi) is defined by N^i-CA^i-Ci-N^{i-1}, and ω (omega) is defined by CA^i-C^i-N^{i+1}-CA^{i+1}; for the protein side chains, χn (chi-n) is defined by C^i-CA^i-CB^i-CG^i.

Cluster 1			
**refp17**	**NHL-a101**	**NHL-a104**	**vp17c2**
Leu8	/	/	/
Leu13	/	/	/
Leu31	/	/	/
Ile34	/	/	/
Val35	/	/	/
Leu85	/	/	/
**Cluster 2**			
**refp17**	**NHL-a101**	**NHL-a104**	**vp17c2**
Ile19	/	/	/
Val88	/	/	/
Ile92	/	/	/
Ile94	Val94	/	/
**Cluster 3**			
**refp17**	**NHL-a101**	**NHL-a104**	**vp17c2**
Leu41	/	/	/
Leu50	/	/	/
Ile60	/	/	/
Leu61	/	/	Ile61
Leu64	/	/	/
Leu68	/	/	/
Leu75	/	/	/
Leu78	/	/	/
Val82	Ile82	/	Met82
Ile104	/	/	/

Supplementary Table S2. Mapping Hydrophobic Clusters. Residues belonging to each HC (cluster 1, cluster 2, and cluster 3) in refp17 and in vp17s are shown. Additionally, mutated residues in vp17s are also indicated.

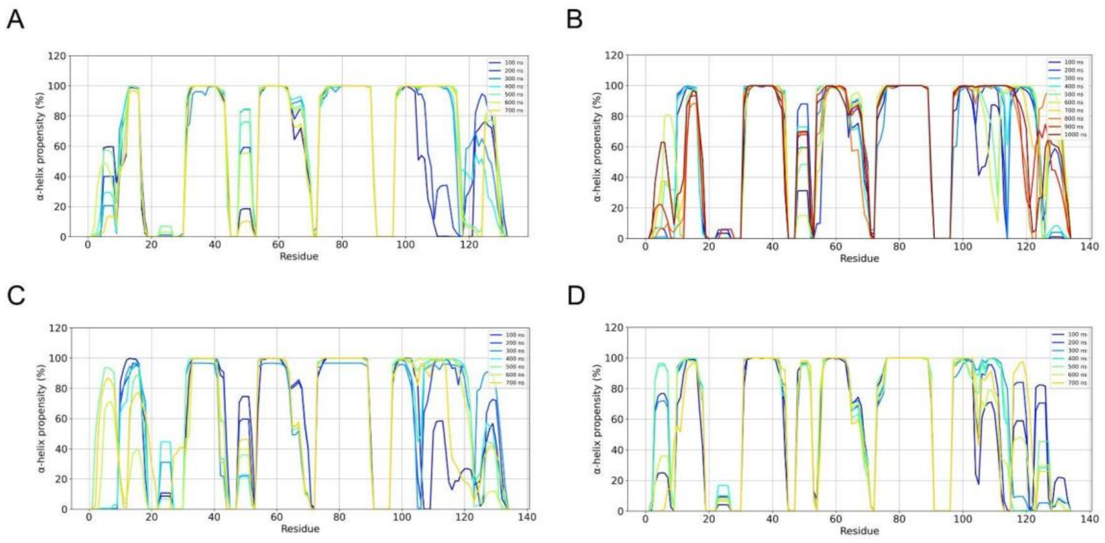

Supplementary Figure S2. Time series of α-helix propensity. (A) α-helix propensity of refp17, (B) NHL-a101, (C) NHL-a104, and (D) vp17c2 are shown for every 100 ns of aMD simulations.

Hydrogen bond interactions					
**Acceptor**	**Donor**	**refp17**	**NHL-a101**	**NHL-a104**	**vp17c2**
THR 53/O	CYS 57/N	91.73%	73.02%	88.28%	1.12%
LYS 27/O	LEU 21/N	90.45%	62.28%	73.08%	71.13%
SER 54/O	ARG 58/N	89.84%	67.65%	90.08% (M)	28.29%
LYS 95/O	ARG 20/N	89.74%	67.80%	5.38%	10.72%
TYR 79/O	ALA 83/N	89.73%	88.58%	92.41%	56.52%
LEU 13/O	GLU 17/N	88.94%	84.29%	61.21%	76.66%
GLU 12/O	TRP 16/N	85.32%	66.03%	59.99% (M)	86.36% (M)
GLU 40/O	PHE 44/N	85.07%	79.75%	86.60%	59.24%
TRP 16/O	ILE 19/N	82.80%	56.92%	30.71%	13.27%
GLU 73/O	SER 77/N	80.67%	72.09%	81.32%	66.32%
SER 72/O	ARG 76/N	80.39%	77.12% (M)	89.31% (M)	69.07% (M)
TRP 36/O	GLU 40/N	79.97%	87.27%	64.05%	74.75%
LEU 41/O	VAL 46/N	79.13%	58.11%	23.95%	7.72%
ILE 19/O	TYR 29/N	76.17%	43.79%	23.58%	2.83%
GLU 99/O	LYS 103/N	75.78%	69.32%	66.71%	85.02%

Supplementary Table S3. Hydrogen bond frequencies of refp17 and vp17s during aMD. Residues involved in the H-bond interaction, as well as the corresponding fraction of time that each interaction lasts, are shown. M, mutated residues.

Hydrophobic interactions					
**Residue 1**	**Residue 2**	**refp17**	**NHL-a101**	**NHL-a104**	**vp17c2**
LEU 8	LEU 13	74.29%	48.57%	54.29%	80.00%
LEU 8	TRP 16	62.86%	54.29%	68.57%	8.57%
LEU 8	LEU 85	60.00%	54.29%	34.29%	8.57%
LEU 13	TRP 16	71.43%	48.57%	40.00%	91.43%
LEU 13	LEU 31	80.00%	82.86%	48.57%	85.71%
LEU 13	ILE 34	71.43%	68.57%	37.14%	14.29%
TRP 16	TYR 29	100.00%	94.29%	71.43%	57.14%
TRP 16	ALA 37	62.86%	45.71%	22.86%	0.00%
TRP 16	VAL 88	88.57%	77.14%	34.29%	88.57%
ILE 19	TYR 29	97.14%	71.43%	54.29%	54.29%
LEU 31	VAL 35	71.43%	82.86%	57.14%	88.57%
TRP 36	ALA 37	62.86%	62.86%	20.00%	42.86%
LEU 41	VAL 46	97.14%	60.00%	94.29%	14.29%
LEU 41	LEU 85	71.43%	71.43%	22.86%	91.43%
PHE 44	VAL 46	88.57%	22.86%	97.14%	11.43%
PHE 44	LEU 75	85.71%	62.86%	42.86%	0.00%
PHE 44	LEU 78	88.57%	65.71%	74.29%	2.86%
LEU 50	ILE 60	91.43%	28.57%	91.43%	2.86%
LEU 51	VAL 82	82.86%	57.14% (M)	80.00%	0.00% (M)
LEU 51	LEU 85	97.14%	80.00%	94.29%	5.71%
LEU 64	LEU 78	94.29%	65.71%	91.43%	82.86%
LEU 75	LEU 78	77.14%	82.86%	45.71%	37.14%
TYR 79	ILE 104	71.43%	85.71%	22.86%	5.71%
LEU 85	VAL 88	60.00%	31.43%	31.43%	51.43%
ILE 92	ILE 94	97.14%	91.43% (M)	68.57%	68.57%
LEU 101	ILE 104	68.57%	65.71%	65.71%	65.71%

Supplementary Table S4. Hydrophobic interactions analysis. comparison of hydrophobic interactions between refp17 and vp17s during aMD. Residues involved in the hydrophobic interaction, as well as the corresponding fraction of time that each interaction lasts, are shown. M, mutated residues.

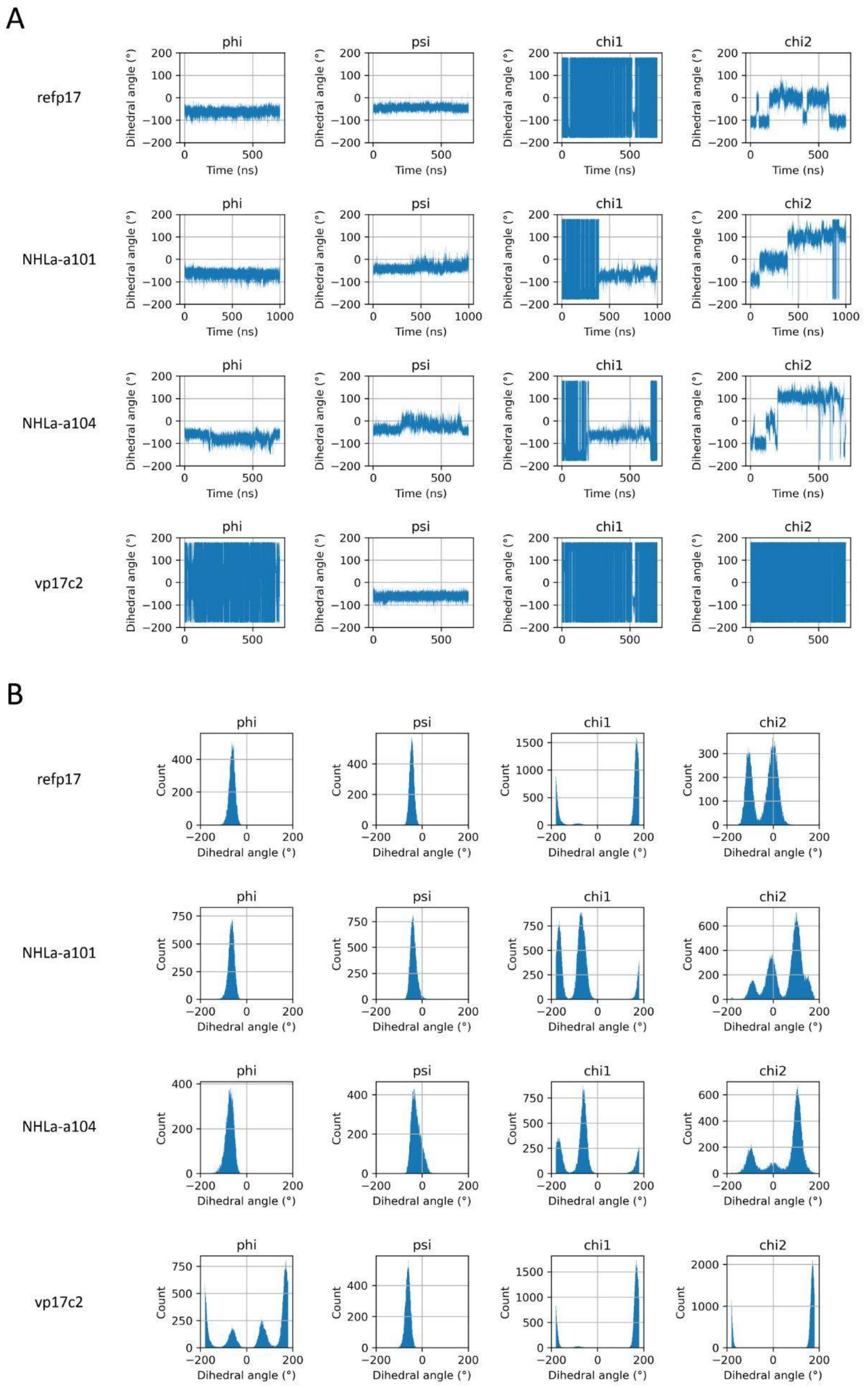

Supplementary Figure S3. Comparative Dihedral Angle Distribution Analysis: (A) Dihedral angle distribution along aMD of phi, psi, chi1, and chi2 dihedral angles of Trp16 in refp17 and vp17s. (B) Density histogram of dihedral angle distribution along aMD of phi, psi, chi1, and chi2 dihedral angles of Trp16 in refp17 and vp17.

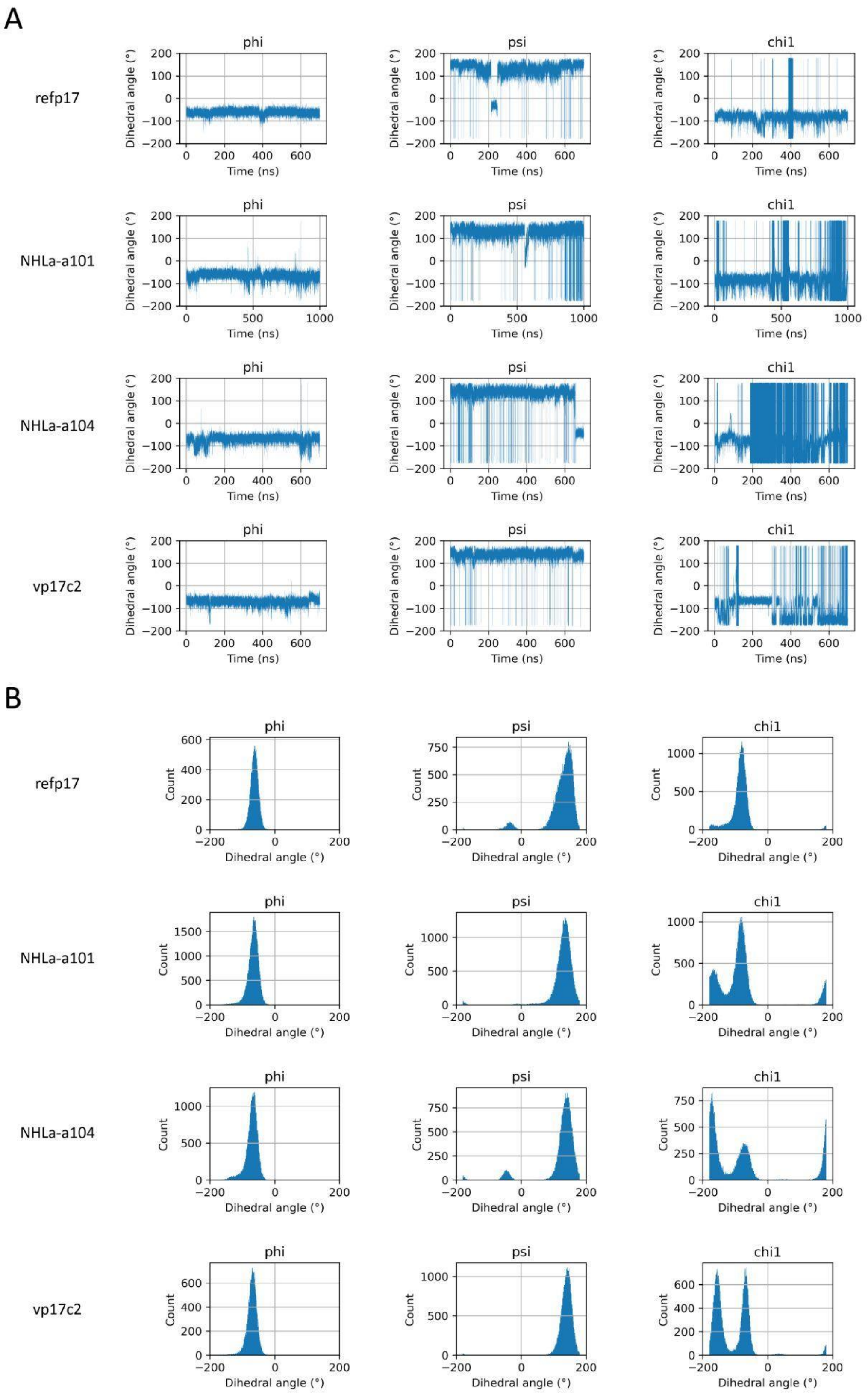

Supplementary Figure S4. Comparative Dihedral Angle Distribution Analysis. (A) Dihedral angle distribution along aMD of phi, psi, and chi dihedral angles of Tyr29 in refp17 and vp17s. (B) Density histogram of dihedral angle distribution along aMD of phi, psi, chi1 dihedral angles of Tyr29 in refp17 and vp17s.

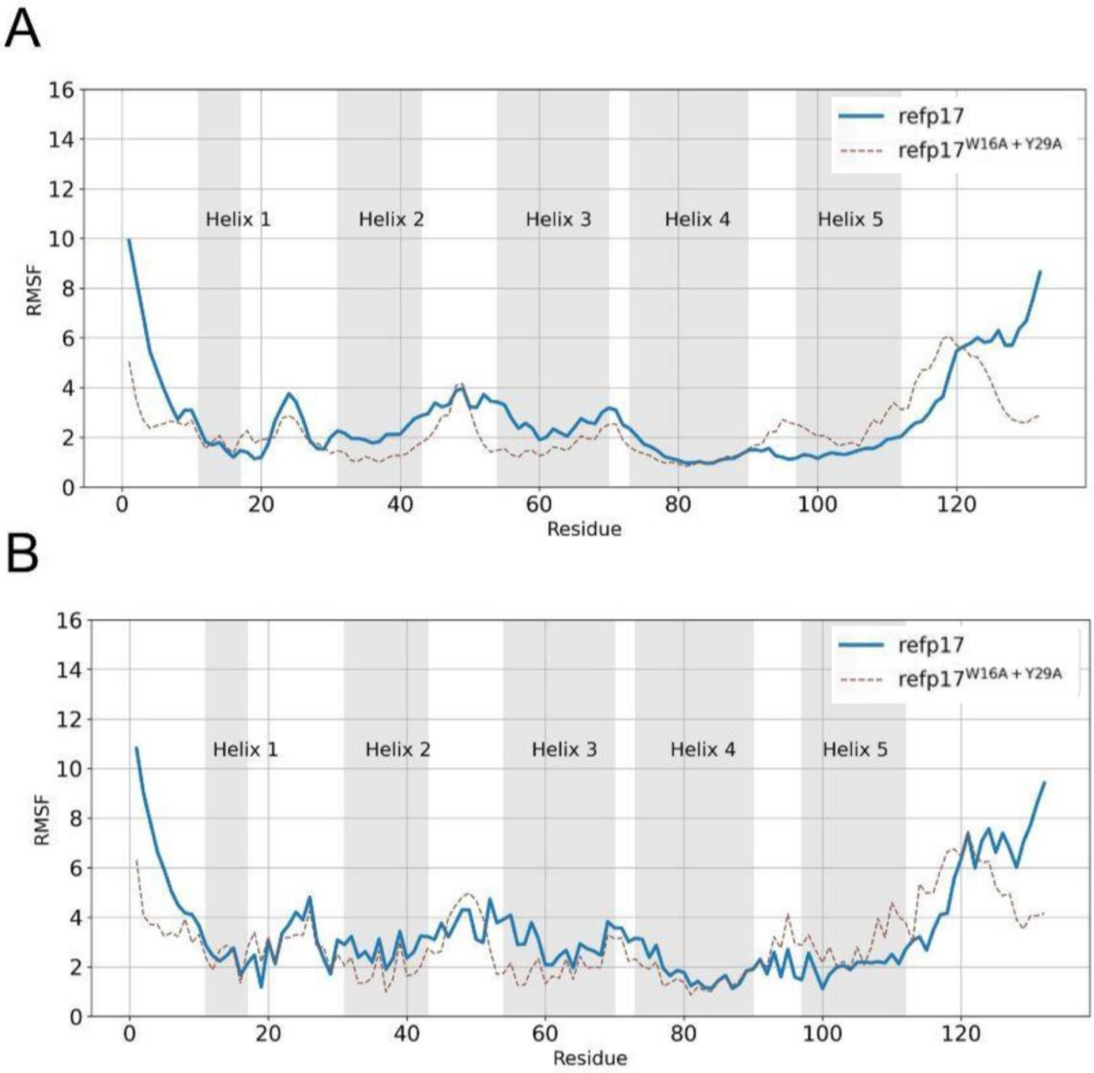

Supplementary Figure S5. Protein Residue Fluctuations analysis. RMSF analysis of the (A) backbone of the protein and (B) of the side chains. The gray regions correspond to the residues forming the α-helices in refp17 [**39**]. The solid blue lines represent fluctuations of refp17 residues, while the dashed brown lines represent the refp17$^{W16A+Y29A}$ protein.

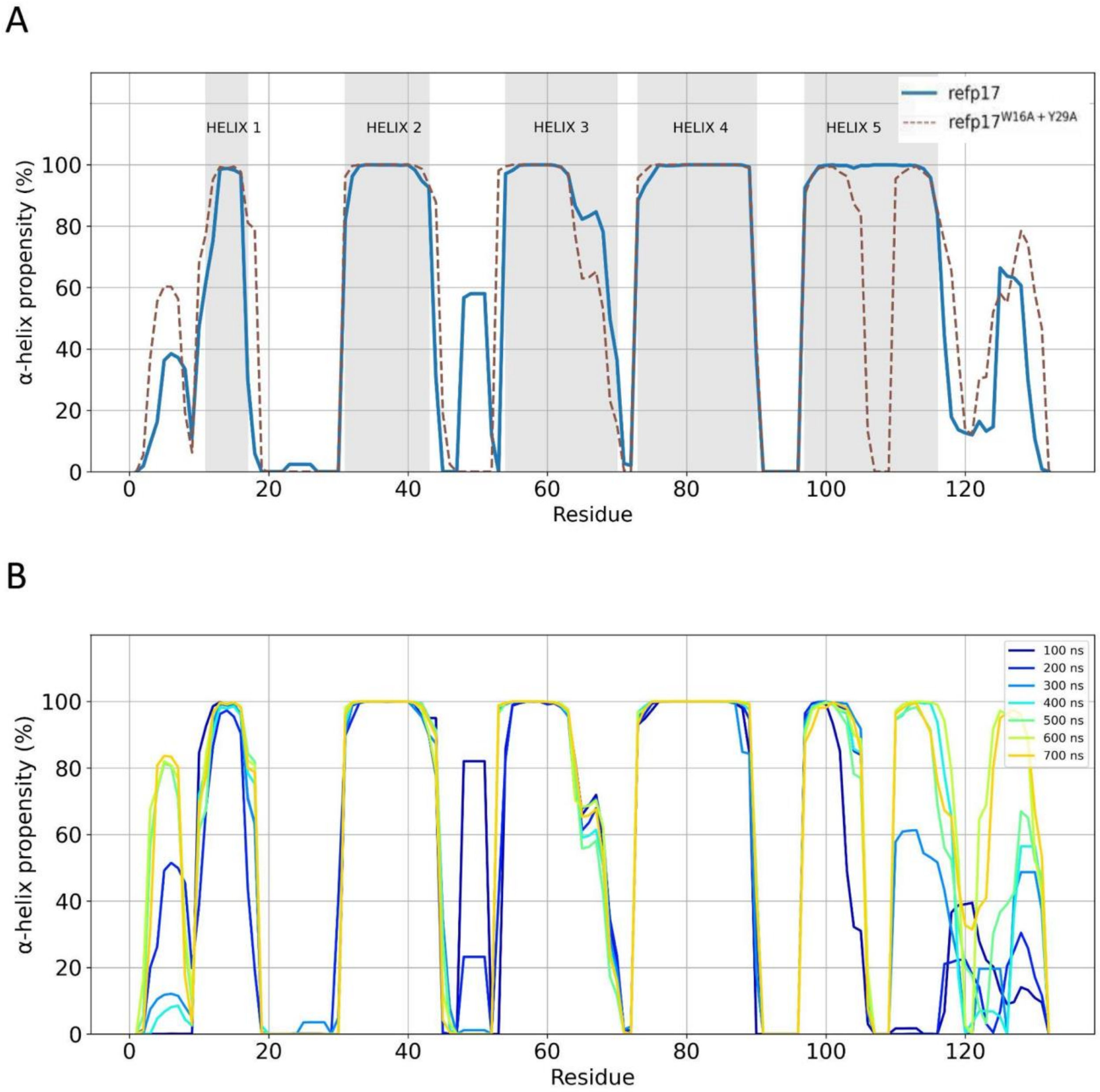

Supplemenatry Figure S6. Alpha helix propensity comparison. (A) Average α-helix propensity profile of refp17$^{W16A+Y29A}$ compared to refp17 from 300ns of aMD. (B) Time series of the refp17$^{W16A+Y29A}$ α-helix propensity every 100 ns step of aMD.

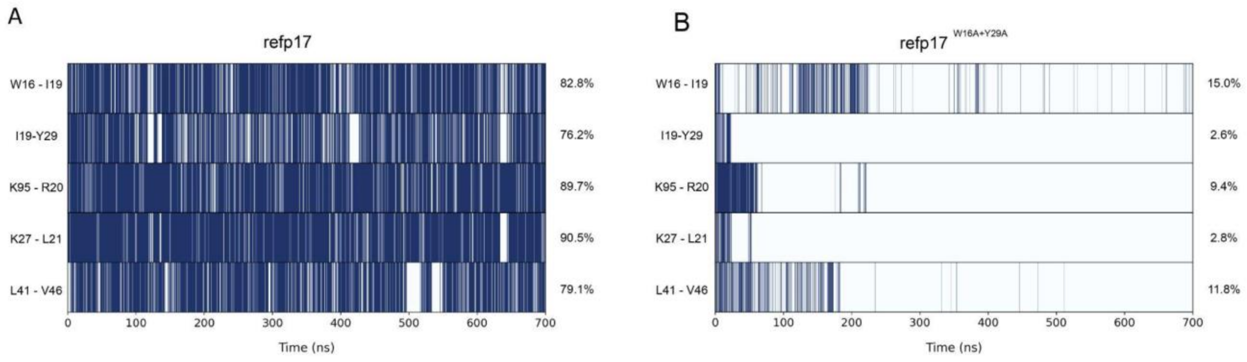

Supplementary Figure S7. Hydrogen bond time series. The analysis finds and tracks H-bonds throughout the aMD simulation. Colors are set according to the presence (Dark blue line) or absence (Light blue line) of the interaction. The analysis compares the refp17$^{W16A+Y29A}$ H-bonds with those of refp17.

Hydrophobic interactions			
Residue 1	***Residue 2***	***refp17***	***refp17$^{W16A+Y29A}$***
LEU 8	*LEU 13*	*74.29%*	*74.29%*
LEU 8	*TRP 16*	*62.86%*	*8.57%* (M)
LEU 8	*LEU 85*	*60.00%*	*34.29%*
LEU 13	*TRP 16*	*71.43%*	*42.86%* (M)
LEU 13	*LEU 31*	*80.00%*	*51.43%*
LEU 13	*ILE 34*	*71.43%*	*82.86%*
TRP 16	*TYR 29*	*100.00%*	*5.71%* (M)
TRP 16	*ALA 37*	*62.86%*	*0.00%* (M)
TRP 16	*VAL 88*	*88.57%*	*88.57%* (M)
ILE 19	*TYR 29*	*97.14%*	*2.86%* (M)
LEU 31	*VAL 35*	*71.43%*	*82.86%*
TRP 36	*ALA 37*	*62.86%*	*34.29%*
LEU 41	*VAL 46*	*97.14%*	*11.43%*
LEU 41	*LEU 85*	*71.43%*	*54.29%*
PHE 44	*VAL 46*	*88.57%*	*11.43%*
PHE 44	*LEU 75*	*85.71%*	*2.86%*
PHE 44	*LEU 78*	*88.57%*	*5.71%*
LEU 50	*ILE 60*	*91.43%*	*0.00%*
LEU 51	*VAL 82*	*82.86%*	*0.00%*
LEU 51	*LEU 85*	*97.14%*	*2.86%*
LEU 64	*LEU 78*	*94.29%*	*94.29%*
LEU 75	*LEU 78*	*77.14%*	*65.71%*
TYR 79	*ILE 104*	*71.43%*	*0.00%*
LEU 85	*VAL 88*	*60.00%*	*42.86%*
ILE 92	*ILE 94*	*97.14%*	*82.86%*
LEU 101	*ILE 104*	*68.57%*	*0.00%*

Supplementary Table S5. Hydrophobic interactions analysis. Comparison of hydrophobic interactions between refp17and refp17$^{W16A+Y29A}$. Residues involved in the hydrophobic interaction, as well as the corresponding fraction of time that each interaction lasts are shown. M, mutated residues.
